# Medication Regimen Complexity and Patient-Reported Adverse Drug Events in Korean Community Pharmacies: A Cross-Sectional Study

**DOI:** 10.3390/pharmacy14010011

**Published:** 2026-01-22

**Authors:** Sunmin Lee, Kyung sun Oh

**Affiliations:** 1 College of Pharmacy and Research Institute of Life and Pharmaceutical Sciences, Sunchon National University, Suncheon 57922, Republic of Korea; smlee@scnu.ac.kr; 2 College of Pharmacy, Yeungnam University, Gyeongsan 38541, Republic of Korea

**Keywords:** medication regimen complexity, MRCI-K, adverse drug events, polypharmacy, patient-reported outcomes

## Abstract

Evidence linking medication regimen complexity to patient-reported adverse drug events (ADEs) is limited. This study examined the association between regimen complexity and patient-reported ADEs among adults using community pharmacy services. A cross-sectional survey was conducted among adults with prescription experience at community pharmacies in Korea (14 January–24 February 2025). Data included MRCI-K scores, medication adherence, ADE reports, comorbidities, polypharmacy status, and demographics. Prescription records verified medication counts and drug-related risks. Determinants of regimen complexity were assessed using multivariable linear regression, and predictors of ADE reporting were examined using multivariable logistic regression. Among 201 participants, 101 (50.2%) reported at least one ADE in the past month. Polypharmacy, comorbidities, and multidose dispensing service use were independently associated with higher regimen complexity, whereas higher income, college education, and older age were associated with lower complexity. Higher MRCI-K scores (OR = 0.95, 95% CI 0.91–0.99) and older age (OR = 0.98, 95% CI 0.96–0.99) were associated with lower odds of ADE reporting. Higher medication regimen complexity and older age were associated with reduced reporting of ADEs, suggesting possible under-recognition among these populations. Patient-centered strategies are needed to enhance ADE identification in individuals with complex medication regimens.

## 1. Introduction

Adverse drug events (ADEs) are defined as any injuries resulting from medication use, irrespective of whether the drug was used appropriately [1]. ADEs include harm caused by adverse drug reactions at therapeutic doses, medication errors, overdoses, and inappropriate use. A systematic review reported that 8–20% of primary care patients experience ADEs, often leading to reduced quality of life, therapeutic failure, and increased healthcare costs [2]. Notably, about one-fifth of ADEs are considered preventable, highlighting the need for systematic monitoring to detect medication-related problems and to minimize harm while optimizing therapeutic outcomes [3].

The number of medications is a major determinant of ADE risk [4]. Polypharmacy—commonly defined as the concurrent use of five or more medications—increases exposure to drug–drug interactions, medication errors, nonadherence, and overall medication regimen complexity, contributing to greater treatment burden and self-management challenges for patients [5]. However, medication count alone cannot fully capture the multifaceted burden of medication use. Regimen complexity represents a distinct dimension of medication burden, such as multiple dosage forms, frequent dosing, and administration instructions, which may challenge patients’ self-management. The Medication Regimen Complexity Index (MRCI), developed by George et al., is a weighted scoring instrument designed to quantify the complexity of pharmacotherapy [6]. It evaluates three domains—dosage forms (Section A), dosing frequency (Section B), and additional administration instructions (Section C)—and combines them into a single composite score. The MRCI provides an objective metric for assessing treatment-related burden, making it a useful tool in both clinical practice and health services research [7].

Higher MRCI scores have been associated with poor adherence, adverse drug reactions, and hospital readmissions across diverse clinical settings [8,9]. A recent meta-analysis further showed that greater medication regimen complexity is significantly associated with increased risks of hospitalization, readmission, and medication nonadherence. However, evidence across studies remains heterogeneous and inconsistent, suggesting that medication regimen complexity alone may not fully explain medication-related risks. A more comprehensive and patient-centered assessment of outcomes has therefore been recommended to advance pharmaceutical care research.

Evidence linking medication regimen complexity with patient behavior–related outcomes, such as adherence and patient-reported ADEs, remains limited. Few studies have addressed this relationship: one found that patients with less complex regimens were more likely to discontinue therapy when feeling worse, while another identified an association between greater complexity, post-discharge medication discrepancies, and adverse drug events or hospitalizations [10,11].

In South Korea, higher medication regimen complexity has been associated with increased reports of adverse drug events among hospitalized patients [12]; however, evidence from outpatient settings remains limited. Although the Korea Adverse Event Reporting System has collected spontaneous adverse drug reaction reports since 1988, reporting remains largely dependent on healthcare professionals, and ADEs occurring in community settings are underreported [13]. To address this gap, evaluating medication regimen complexity as an indicator of patient burden and examining its association with patient-reported ADEs are essential. Furthermore, identifying determinants of high regimen complexity is necessary to establish an evidence-based framework for screening target populations in community pharmacy practice.

Given these gaps, this study aimed to (1) identify determinants of medication regimen complexity and (2) examine the association between medication regimen complexity and patient-reported ADEs among adults receiving prescription medications in community pharmacies in Korea.

## 2. Materials and Methods

### 2.1. Design and Setting

This cross-sectional survey was conducted between 14 January and 24 February 2025, by two trained researchers with academic backgrounds in pharmacy, under the supervision of the principal investigator. To ensure consistency, all researchers received prior training and adhered to standardized data collection protocols across community pharmacies in Korea. The survey was conducted at four main community pharmacies located near the largest general hospital in eastern Jeonnam Province, a 600-bed tertiary-care hospital. These pharmacies provide both prescription dispensing and over-the-counter medication services. Eligible participants were adults aged 20 years or older with prior experience of prescription dispensing at community pharmacies and the cognitive, emotional, and physical ability to complete the survey independently. Patients who did not provide consent, used proxy prescription services, or were unable to self-report were excluded. Participants were recruited through convenience and snowball sampling. Data were collected using a self-administered questionnaire that included the Korean version of the Adherence to Refills and Medications Scale (ARMS-K), questions on patient-level medication experiences and self-recognized potential adverse drug events, and demographic and clinical characteristics. Prescription records were reviewed to assess the number of medications and regimen complexity. All responses and prescription data were cross-checked and verified by two independent researchers for accuracy and consistency.

### 2.2. Data Collection

#### 2.2.1. Medication Regimen Complexity Index (MRCI-K)

Regimen complexity was assessed using the Korean version of the Medication Regimen Complexity Index (MRCI-K) [12]. This tool evaluates three domains: formulation, dosing frequency, and administration instructions [6]. Each domain is weighted according to regimen complexity, and the summed score represents overall medication regimen complexity, with higher scores indicating greater complexity. The number of prescribed medications was verified from prescription records.

#### 2.2.2. Adherence to Refills and Medications Scale (ARMS-K)

Medication adherence was measured with the validated Korean version of the Adherence to Refills and Medications Scale (ARMS-K) [14]. This instrument consists of a total of 12 items, including eight items assessing adherence to medication-taking behaviors and four items assessing adherence to prescription refills. Each item is rated on a 4-point Likert scale with response options ranging from “never,” “sometimes,” “often,” to “always.” Responses are summed to yield a total score, with lower scores indicating better medication adherence. Internal consistency was acceptable, with Cronbach’s alpha of 0.80 in the original validation study and 0.70 in the present study.

#### 2.2.3. Patient-Reported Adverse Drug Events (ADEs)

The ADE items were adapted from the Patient-Reported Outcome Measure, Inquiry into Side Effects (PROMISE) [15], which originally included the presence of predefined symptoms and drug-relatedness. To enhance epidemiological relevance to Korean patients, the list was modified to reflect the 12 most frequently reported symptoms in the 2023 Korean pharmacovigilance database, which included nausea, headache, dizziness, myalgia, urticaria, pruritus, vomiting, rash, fever, diarrhea, dyspnea, and chest discomfort, as well as one open-ended item. Participants who reported a symptom and indicated that it was related to their medication were classified as having experienced an ADE within the past month, whereas those who assessed their symptoms as unrelated to medication use were excluded from the ADE report group.

#### 2.2.4. Covariates

Covariates included the number of self-reported comorbidities from a predefined list of 30 conditions [16]; polypharmacy, defined as the use of five or more regular medications; and the number of regularly prescribed drugs. Additional covariates included sex, age, income, perceived medication cost burden, assessed as a patient-reported measure reflecting the individual’s perceived financial burden of medication costs, insurance type, and use of a multidose dispensing service, defined as a community pharmacy-provided system in which multiple medications are prepared and dispensed in dose-specific bags according to scheduled dosing times [17] and prior receipt of medication education from healthcare professionals.

### 2.3. Statistical Analysis

Demographic and clinical characteristics were summarized according to adverse drug event status. A Pearson correlation analysis was conducted to evaluate the linear associations between the main continuous variables of the study: regimen complexity, age, number of comorbidities, and medication adherence. Multivariable linear regression was conducted to examine determinants of medication regimen complexity, with the MRCI-K score treated as a continuous dependent variable. Model assumptions were verified by residual analysis. Logistic regression was used to identify independent predictors of patient-reported ADEs. Multicollinearity was assessed using variance inflation factors, tolerances, and condition indices. To evaluate the robustness of our findings with respect to polypharmacy, two sensitivity analyses were conducted. First, a parsimonious model excluding polypharmacy was fitted. Second, polypharmacy was replaced by the total number of medications in the multivariable model. Statistical significance was defined as *p* < 0.05. All analyses were performed using Python (version 3.10).

## 3. Results

A total of 201 patients were included, and 101 (50.2%) reported at least one patient-reported ADE during the past month. Table 1 presents the baseline characteristics of the study population and compares patients with and without ADEs. The mean age of participants was 64.4 years (SD 15.6), and 102 (50.7%) were male. No statistically significant differences were observed between the two groups in baseline demographic or clinical characteristics, including age, sex, MRCI-K score, perceived medication cost burden, and polypharmacy status. No statistically significant difference in polypharmacy prevalence was observed between groups (60.4% vs. 49.0%, *p* = 0.093).

Table 2 summarizes the Pearson correlation coefficients among key study variables. The MRCI-K score was strongly correlated with the total number of medications (r = 0.869, *p* < 0.001) and correlated with the number of comorbidities (r = 0.292, *p* < 0.05). No significant correlations were observed between MRCI-K and age or medication adherence.

Multivariable linear regression identified several independent predictors of medication regimen complexity (Table 3). Polypharmacy was the strongest determinant, associated with a 12.97-point higher MRCI-K score (95% CI 10.93–15.02, *p* < 0.001). The number of comorbidities (β = 0.95, 95% CI 0.14–1.75, *p* = 0.022) and the use of a multidose dispensing service (β = 3.04, 95% CI 0.75–5.34, *p* = 0.01) were also positively associated with complexity. In contrast, higher income (β = −2.95, 95% CI −5.38 to −0.52, *p* = 0.018), college or higher education (β = −3.37, 95% CI −5.51 to −1.23, *p* = 0.002), and older age (β = −0.07 per year, 95% CI −0.15 to −0.00, *p* = 0.044) were significantly associated with lower complexity. Sex, living alone, cost burden, medication education, and insurance type were not significantly related to MRCI-K scores.

Univariable logistic regression showed no significant associations between most covariates and ADE reporting (Table 4). In the multivariable analysis, higher MRCI-K scores were significantly associated with lower odds of reporting an ADE (OR = 0.95, 95% CI 0.91–0.99, *p* = 0.024). Older age was also associated with decreased reporting (OR = 0.98 per year, 95% CI 0.96–0.99, *p* = 0.029). The number of comorbidities showed a positive but borderline association with ADR awareness (OR = 1.27, 95% CI 0.99–1.63, *p* = 0.065). Other covariates, including polypharmacy, sex, medication cost burden, dispensing setting, and ARMS-K score, were not significantly associated with ADR awareness. The associations of MRCI-K and age with ADE remained significant after excluding polypharmacy and when substituting the total number of medications for the binary polypharmacy variable. Estimates were consistent across models, indicating that the findings were robust and independent of polypharmacy definitions (Supplementary Table S1).

## 4. Discussion

This study is the first investigation to examine the relationship between medication regimen complexity, measured by the MRCI-K, and patient-reported potential ADEs within community pharmacy settings in Korea. Regimen complexity itself increased with a higher number of medications, greater comorbidities, and the use of a multidose dispensing service, but decreased with higher income, higher education, and older age. The results showed that higher medication regimen complexity was associated with lower odds of patient-reported ADEs, independent of polypharmacy and other clinical factors. These findings indicate that higher regimen complexity was not associated with greater patient recognition of ADEs in community pharmacy settings, suggesting a possible discrepancy between objective medication regimen complexity and patient-reported potential ADEs.

MRCI-K scores showed a positive correlation with both the number of prescribed medications (r = 0.869) and the number of comorbidities, showing that polypharmacy remains the major determinant of regimen complexity in patients. This finding is consistent with previous studies reporting a similar relationship between medication count, multimorbidity, and regimen complexity [18]. Polypharmacy showed the strongest association with MRCI-K scores (β = 12.97, 95% CI 10.93–15.02), supporting evidence from primary care settings that identified the number of medications as the major contributor to regimen burden. The use of multidose dispensing services was also identified as an independent factor associated with higher complexity, reflecting the clustering of patients with extensive medication use who require pharmaceutical management [19]. In contrast, no significant association was observed between medication regimen complexity and medication adherence, unlike previous studies reporting an inverse relationship [20]. This suggests that adherence may be influenced by factors other than medication regimen complexity. In Korea, the adoption of multidose dispensing systems is common, particularly among patients with multiple chronic conditions (n = 124, 61.7%). Higher socioeconomic status—reflected by higher income and education levels—and older age were also associated with lower regimen complexity. These findings suggest that medication regimen complexity is influenced by multiple determinants, encompassing disease burden, medication load, and social factors. Further research is needed to identify specific modifiable factors that can be targeted through pharmacist-led interventions to optimize medication management.

Higher MRCI-K scores and older age were independently associated with lower odds of patient-reported ADEs after adjusting for multiple demographic and clinical factors, including polypharmacy. This inverse association suggests that greater regimen complexity may be linked to reduced recognition of drug-related harm. Notably, this finding contrasts with previous studies reporting that older age, polypharmacy, and multimorbidity are positively associated with ADR reporting [21,22]. Although polypharmacy may increase the risk of inappropriate prescribing and drug–drug interactions among older adults [5], patients managing highly complex medication regimens may have a reduced ability to recognize or report adverse drug effects. Multimorbid patients with higher regimen complexity may attribute subtle symptoms to their underlying diseases or aging rather than to medication use, leading to underreporting [23]. Managing complex medication also requires significant cognitive and organizational effort; thus, patients may adhere to schedules over monitoring changes. These findings suggest that higher regimen complexity may impair pharmacovigilance awareness and symptom recognition among community-dwelling adults. Our study evaluated patient-reported events within a community pharmacy context, where spontaneous recognition and subjective symptom attribution play a critical role, unlike hospital-based studies that focused on clinically confirmed or severe ADEs. This outcome likely reflects the multifaceted nature of ADE recognition, where medication regimen complexity, cognitive burden, and symptom ambiguity collectively influence patient awareness and reporting behavior [24].

In addition, previous studies have generally associated higher MRCI scores with increased risks of adverse outcomes such as hospitalizations and bleeding-related admissions [25,26]. The discrepancy between these findings and ours may reflect differences in outcome definitions. While these studies focused on objectively assessed clinical events, the present study assessed patient-reported symptoms that reflect patients’ subjective recognition and attribution. These results highlight a dimension of pharmacovigilance: medication regimen complexity may influence patients’ perceptual and behavioral responses to them. This underscores the need to distinguish between the occurrence and recognition of ADEs when evaluating medication safety in community settings.

In Korea, over 70% of pharmacists practice in community pharmacies, which are highly accessible and function as the first contact for medication-related care. After the separation of prescribing and dispensing, community pharmacies became predominantly located near clinics or hospitals and primarily focused on dispensing medications [27,28]. In routine practice, community pharmacists perform medication safety checks using the national Drug Utilization Review (DUR) system, which is integrated into pharmacy dispensing software and provides alerts for contraindications, age or pregnancy restrictions, therapeutic duplication, and drug–drug interactions [28].

Pharmacists’ participation in adverse drug reaction reporting is considered a critical component of pharmacovigilance and patient safety. However, a survey showed that although approximately 87% of Korean community pharmacists had encountered ADR cases in their practice, only 29.4% had submitted an ADR report [29], highlighting a gap between ADR recognition and reporting. This underscores the need to strengthen patient-centered pharmacovigilance approaches and to support community pharmacists in reporting patient-experienced adverse drug events.

Our study suggests the importance of pharmacist engagement with patients who have high regimen complexity [30]. Patients with highly complex regimens may require targeted counseling and structured follow-up to enhance awareness of potential adverse effects. In addition to score-based assessments, pharmacists should provide patient-centered counseling to address medication burden and discuss potential adverse effects associated with complex regimens. Our findings suggest that complementary strategies are needed to enhance the detection and management of mild-to-moderate, patient-experienced ADEs at the community level.

This study was conducted in real-world community pharmacy settings, employed validated instruments, and applied multivariable regression models with sensitivity analyses excluding and redefining polypharmacy to verify robustness. Nevertheless, several limitations should be acknowledged. First, ADEs were assessed through patient self-reports, which may be affected by recall bias and symptom misattribution. However, this method captures subjective experiences and patient perspectives, information often absent from clinical records but essential for designing patient-centered pharmaceutical care [24]. Second, the study did not include clinical verification of ADE severity or causality, which limits interpretation regarding clinical significance. Third, although only self-report–capable participants were included, age-related cognitive decline or low medication literacy may still have impaired symptom recognition. Fourth, the cross-sectional design and recruitment from a limited number of community pharmacies may, within a single geographic region, affect the generalizability of the findings. In addition, convenience sampling may have introduced selection bias, as this exploratory study relied on voluntary participation to capture patient experiences in routine community pharmacy practice. Finally, participants reported all medications they were taking, including OTC products and supplements, but self-reported lists may still have been incomplete, potentially underestimating regimen complexity.

Future multicenter studies with representative populations are required to confirm these findings. Research on pharmacist-led counseling interventions is warranted to identify target patient groups and to determine whether interventions can enhance ADE recognition and reporting among individuals with high regimen complexity for the development of patient-centered medication management systems in community pharmacy practice.

## 5. Conclusions

Medication regimen complexity was strongly associated with the number of medications, with polypharmacy as the primary determinant. Higher regimen complexity and older age were associated with lower odds of patient-reported adverse drug events after adjustment for polypharmacy. These findings suggest a gap between objective regimen complexity and patients’ recognition and reporting of adverse drug events in community pharmacy settings in Korea, indicating the potential importance of strategies to support patient awareness and monitoring in community medication management.

## Figures and Tables

**Table 1:** Baseline demographic and clinical characteristics of participants by adverse drug event status (*n* = 201).

Characteristic		Total (N = 201)	Adverse Drug Event: No (N = 100)	Adverse Drug Event: Yes (N = 101)	*p*-Value
Age, mean (SD)		64.4 (15.6)	65.4 (14.9)	63.4 (16.3)	0.311
Sex, male, n (%)		102 (50.7)	54 (54.0)	48 (47.5)	0.457
Education level, n (%)					0.817
	High school or less	105 (52.2)	53 (53.0)	52 (51.5)	
	College or more	96 (47.8)	47 (47.0)	49 (48.5)	
Income level, n (%)					0.631
	<3 million KRW	74 (36.8)	39 (39.0)	35 (34.7)	
	≥3 million KRW	127 (63.2)	61 (61.0)	66 (65.3)	
Perceived medication cost burden, n (%)		75 (37.3)	37 (37.0)	38 (37.6)	0.835
National insurance, n (%)		148 (73.6)	70 (70.0)	78 (77.2)	0.202
Living alone, n (%)		33 (16.4)	17 (17.0)	16 (15.8)	0.835
Medication education, n (%)		132 (65.7)	66 (66.0)	66 (65.3)	0.631
Multidose dispensing service, n (%)		124 (61.7)	59 (59.0)	65 (64.4)	0.471
Num comorbidities, mean (SD)		1.9 (1.8)	1.8 (1.8)	2.0 (1.9)	0.448
Polypharmacy (≥5 drugs), n (%)		110 (54.7)	49 (49.0)	61 (60.4)	0.093
Total medications, mean (SD)		4.65 (2.5)	4.80 (2.72)	4.51 (2.4)	0.427
MRCI-K, mean (SD)		16.5 (11.0)	17.5 (11.8)	15.5 (10.1)	0.203
ARMS-K score, mean (SD)		43.1 (5.2)	42.8 (5.0)	43.3 (5.4)	0.509

Abbreviations: ARMS-K = Adherence to Refills and Medications Scale—Korean; MRCI-K = Medication Regimen Complexity Index—Korean.

**Table 2:** Pearson correlation coefficients (r) among key study variables (*n* = 201).

Variable	MRCI-K	Total Medications	Comorbidities	Age	ARMS-K Score
MRCI-K	1	0.869 ***	0.292 *	0.124	0.100
Total medications		1	0.282 *	0.187 **	0.090
Comorbidities			1	0.434 *	0.108
Age				1	0.116
ARMS-K score					1

ARMS-K = Adherence to Refills and Medications Scale—Korean; MRCI-K = Medication Regimen Complexity Index—Korean * *p* < 0.05; ** *p* < 0.01; *** *p* < 0.001.

**Table 3:** Multivariable linear regression analysis of factors associated with medication regimen complexity (MRCI-K score, *n* = 201).

Variable	Coefficient (β)	95% CI	*p*-Value
Polypharmacy (ref. No)	12.97	[10.93–15.02]	<0.001
Num Comorbidities	0.95	[0.14–1.75]	0.022
Multidose dispensing (ref. None)	3.04	[0.75–5.34]	0.01
Income (ref. <3 M KRW)	−2.95	[−5.38–−0.52]	0.018
Education (ref. ≤ High School)	−3.37	[−5.51–−1.23]	0.002
Age	−0.07	[−0.15–−0.00]	0.044
Sex (ref. Male)	0.08	[−1.97–2.12]	0.941
Living alone (ref. No)	−2.27	[−4.98–0.45]	0.101
Medication cost burden (ref. No)	−1.66	[−3.90–0.58]	0.145
Medication education (ref. No)	−0.22	[−2.31–1.87]	0.839
Insurance (ref. National)	−0.37	[−3.65–2.92]	0.825

**Table 4:** Univariable and multivariable logistic regression analysis of factors associated with adverse drug event (ADE) reporting.

Variable	Univariable OR (95% CI)	*p*-Value	Multivariable OR (95% CI)	*p*-Value
MRCI-K	0.98 (0.95–1.01)	0.13	0.95 (0.91–0.99)	0.024 *
Age	0.99 (0.97–1.01)	0.179	0.98 (0.96–1.00)	0.029 *
Number of comorbidities	1.09 (0.90–1.33)	0.364	1.27 (0.99–1.63)	0.065
Polypharmacy	0.98 (0.56–1.72)	0.956	1.80 (0.78–4.14)	0.167
Sex	0.57 (0.32–1.02)	0.06	0.58 (0.31–1.07)	0.082
Medication cost burden	1.04 (0.58–1.84)	0.902	0.89 (0.48–1.68)	0.727
Multidose dispensing	0.64 (0.33–1.24)	0.187	0.75 (0.37–1.50)	0.415
ARMS-K score	1.04 (0.96–1.12)	0.359	1.05 (0.97–1.14)	0.268

ARMS-K = Adherence to Refills and Medications Scale—Korean; MRCI-K = Medication Regimen Complexity Index—Korean; * *p* < 0.05.

## Data Availability

The original contributions presented in this study are included in the article/Supplementary Material. Further inquiries can be directed to the corresponding author.

## Supplementary Materials

The following supporting information can be downloaded at: https://www.mdpi.com/article/10.3390/pharmacy14010011/s1, Table S1: Correlation matrix, mean, and standard deviations of the 14 items.
