# Supplementary material for: Medication Regimen Complexity and Patient-Reported Adverse Drug Events in Korean Community Pharmacies: A Cross-Sectional Study
Source: Pharmacy (Basel). 2026 Jan 22;14(1):11. doi: 10.3390/pharmacy14010011 (PMC12921983; doi:10.3390/pharmacy14010011)
Supplement: Supplementary file 1 [file pharmacy-14-00011-s001.zip › pharmacy-4056111-supplementary.pdf]

Supplementary Table 1. Correlation matrix, mean and standard deviations of the 14 items.

	Item 1	Item 2	Item 3	Item 4	Item 5	Item 6	Item 7	Item 8	Item 9	Item 10	Item 11	Item 12	Item 13	Item 14
**Item 1**	1.000													
**Item 2**	0.732*	1.000												
**Item 3**	0.686*	0.714*	1.000											
**Item 4**	0.638*	0.694*	0.795*	1.000										
**Item 5**	0.398*	0.377*	0.422*	0.448*	1.000									
**Item 6**	0.425*	0.408*	0.549*	0.628*	0.712*	1.000								
**Item 7**	0.646*	0.664*	0.646*	0.669*	0.568*	0.661*	1.000							
**Item 8**	0.515*	0.562*	0.632*	0.583*	0.376*	0.550*	0.703*	1.000						
**Item 9**	0.461*	0.482*	0.599*	0.664*	0.449*	0.610*	0.654*	0.754*	1.000					
**Item 10**	0.368*	0.371*	0.547*	0.576*	0.464*	0.612*	0.525*	0.632*	0.715*	1.000				
**Item 11**	0.338*	0.372*	0.559*	0.565*	0.459*	0.578*	0.563*	0.607*	0.694*	0.746*	1.000			
**Item 12**	0.314*	0.322*	0.515*	0.535*	0.426*	0.572*	0.534*	0.611*	0.665*	0.778*	0.849*	1.000		
**Item 13**	0.446*	0.441*	0.463*	0.492*	0.634*	0.680*	0.641*	0.632*	0.646*	0.633*	0.617*	0.603*	1.000	
**Item 14**	0.399*	0.419*	0.447*	0.438*	0.539*	0.585*	0.606*	0.602*	0.615*	0.527*	0.521*	0.567*	0.779*	1.000
**Mean**	1.590	1.565	1.655	1.630	1.335	1.365	1.560	1.580	1.805	1.655	1.540	1.550	1.440	1.465
**SD**	1.101	0.969	1.059	0.999	0.706	0.744	0.980	0.960	1.064	1.059	0.986	1.011	0.812	0.945

Note. *$p < .0001$
